# Hydrogels in Simulated Microgravity: Thermodynamics at Play

**DOI:** 10.3390/gels11050342

**Published:** 2025-05-03

**Authors:** Azadeh Sepahvandi, Joseph Johnson, Ava Arasan, Ryan Cataldo, Seyed Majid Ghoreishian

**Affiliations:** 1 Department of Mechanical Engineering, University of South Carolina, Columbia, SC 29201, USA; jmj22@email.sc.edu (J.J.); rcataldo@email.sc.edu (R.C.); 2 Davis College of Engineering, University of California, Davis, CA 95616, USA; akarasan@ucdavis.edu; 3 Center for Energy and Environmental Solutions (CEES), College of STEM-T, South Carolina State University, Orangeburg, SC 29117, USA; sghoreis@scsu.edu

**Keywords:** hydrogel, simulated microgravity, thermodynamics, material behavior, swelling dynamics, crosslinking mechanisms

## Abstract

Hydrogels have become indispensable in biomedical research and regenerative therapies due to their high water content, tissue-like mechanics, and tunable biochemical properties. However, their behavior under altered gravitational conditions—particularly simulated microgravity (SMG)—presents a frontier of challenges and opportunities that remain underexplored. This comprehensive review provides a detailed comparative analysis of hydrogel performance in normal gravity versus SMG environments, focusing on the structural, physicochemical, and thermodynamic parameters that govern their functionality. We critically examine how microgravity influences polymer network formation, fluid dynamics, swelling behavior, mechanical stability, and degradation kinetics. SMG disrupts convection, sedimentation, and phase separation, often leading to inhomogeneous crosslinking and altered diffusion profiles. These changes can compromise hydrogel uniformity, anisotropy, and responsiveness, which are essential for biomedical applications such as drug delivery, tissue regeneration, and biosensing. To address these limitations, we propose a thermodynamic framework that integrates osmotic pressure regulation, entropy-driven swelling, and pressure–temperature control to enhance hydrogel stability and functionality in low-gravity environments. The integration of predictive modeling approaches—including finite element simulations, phase-field models, and swelling kinetics—provides a robust pathway to design space-adapted hydrogel systems. The review also outlines future directions for optimizing hydrogel platforms in extraterrestrial settings, advocating for synergistic advances in material science, biophysics, and space health. These insights offer a strategic foundation for the rational development of next-generation hydrogel technologies tailored for long-duration space missions and planetary biomedical infrastructure.

## 1. Introduction

**Figure 1:**
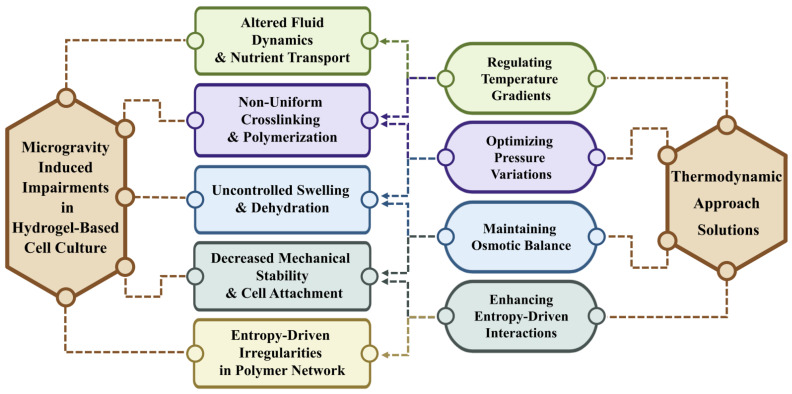
Microgravity-induced impairments in hydrogel-based cell culture (**left**) and their resolution through thermodynamic parameters (**right**). Challenges such as altered fluid dynamics, non-uniform crosslinking, and mechanical instability are mitigated by regulating temperature, pressure, osmotic balance, and entropy-driven interactions.

Hydrogels are three-dimensional polymeric networks that have demonstrated immense potential in tissue engineering and regenerative medicine, offering a biomimetic extracellular matrix that supports cell adhesion, proliferation, and differentiation [1]. Their ability to retain large amounts of water and provide tunable mechanical and biochemical properties, largely through controlling swelling behavior, makes them invaluable for applications in drug delivery, wound healing, and organoid development [2]. Research into hydrogel applications continues to progress, and scientists have begun to employ novel experimental conditions to improve and overcome limitations in hydrogel function; one such solution is the implementation of SMG [1]. As aerospace engineering develops and humans continue investigating space, research regarding how deviations from Earth’s gravity influence human micro and macro physiology has advanced. Microgravity, a state in which an object experiences minimal gravitational force, can incur distinct behaviors in significant biological processes such as differentiation, diffusion, and cellular response [3,4]. The integration of hydrogels with SMG has further expanded their potential, as microgravity-induced alterations in fluid dynamics, mass transport, and mechanical forces can influence hydrogel network formation and crosslinking efficiency [4]. Studies have shown that SMG enhances tissue formation by promoting uniform cell distribution, improving diffusion, and reducing shear-induced mechanical stress, making hydrogel-based cultures in SMG particularly advantageous for space medicine and tissue engineering research [5]. Hydrogels consist of several hydrophilic functional groups, allowing them to retain fluid, nutrients, and solutes. Their consequent microenvironment makes them excellent candidates for performing drug delivery and tissue regeneration mechanisms [6]. Hydrogels influence solute diffusion via lattice spacing: particles experience more steric force when passing through finer mesh versus wider mesh patterns [7]. Additionally, when diffusion occurs under Earth’s conditions, gravity has a lesser effect on smaller particles, which will remain suspended and in motion, while larger molecules will sediment first, a phenomenon known as Brownian motion [8]. As past experimentation has shown, sedimentation can disrupt the passage of cells and molecules, causing them to accumulate in unpredictable regions and ways [9]. Under microgravity, all molecules experience random Brownian motion, circumventing sedimentation and diffusing homogeneously. Additionally, cell cultures conducted under microgravity have demonstrated benefits such as improved cellular aggregation and enhanced overall tissue regeneration. In recent years, scientists have begun to transition from 2D cell cultures due to limitations in cell-cell interactions, lack of an appropriate tissue microenvironment, and cell morphology [10,11]. Within 3D hydrogel cell structures, cells grow within an environment more akin to natural tissue. They can gather into spheroids, spherical groupings of microtissue that can be layered and arranged into tissues and organoids [1,12]. In order to ensure the stability of and control the formation of new tissues, scientists can engineer hydrogels’ mesh patterns and pore sizes. Larger pore sizes allow for unhindered, uniform diffusion of cell growth medium to aggregates. Smaller pore sizes create improved structural support for forming tissues; however, the survivability of these cells is reduced because dense polymer packing physically and chemically prevents nutrients from perfusing to cells [7,13,14,15]. Under microgravity, cells are subject to less gravitational force and require less structural support, reducing the need and risks associated with denser hydrogels [15]. Ultimately, cells grown under microgravity do not experience gravitational stress, sedimentation, or buoyancy gradients, allowing for more efficient tissue aggregation [15,16,17]. Experiments conducted in microgravity help scientists more easily isolate, manipulate, and study control factors altering the hydrogel system’s behavior [8]. Ultimately, understanding these phenomena can help maintain biological functions in space as well as improve health and regenerative medicine on Earth [5,18]. Despite these advantages, hydrogels in SMG exhibit unique challenges that can hinder their structural stability and functionality. The absence of buoyancy-driven convection disrupts polymerization kinetics, leading to inconsistencies in crosslinking density and phase separation [18]. Additionally, altered osmotic pressure gradients, ionic transport, and entropy-driven swelling responses impact the equilibrium behavior of hydrogels, affecting their mechanical properties, degradation rates, and overall functionality [14]. Understanding and mitigating these effects are critical for optimizing hydrogel performance in microgravity-based regenerative therapies and developing standardized experimental procedures. While numerous studies have explored hydrogel behavior in altered gravity conditions, the experimental results remain difficult to compare due to distinct experimental conditions and research focus. Along with their many applications, both differences in hydrogel preparation methods and their surroundings’ chemical properties directly influence the hydrogel’s behavior. Significant performance indicators such as swelling and stiffness are influenced by the selected polymers, the concentration of hydrophilic to hydrophobic polymers, and processing specifications [1,6]. Additionally, although microgravity studies have been conducted since space missions have been viable, studies regarding SMG on regenerative medicine remain relatively niche. While some research teams focus more on SMG’s impact on hydrogels themselves, others more directly study cell behavior in SMG, using hydrogels as a support rather than a focus. Hydrogel’s versatility allows for distinctive research; however, the resulting specificity generates challenges in discovering identifiable trends in hydrogel behavior under SMG [1,2]. Despite these obstacles, a thermodynamic approach offers a promising strategy to stabilize hydrogel behavior under SMG conditions as the hydrogel’s reaction to fluctuations in pH, heat, and concentration largely influence the viability and efficiency of cell cultures [19]. Key thermodynamic parameters, including temperature gradients, pressure variations, osmotic balance, and molecular diffusion, govern phase transitions, network relaxation, and solute transport within hydrogel matrices. By precisely tuning these variables, it is possible to enhance hydrogel swelling dynamics, regulate crosslinking efficiency, and ensure mechanical stability, making them more adaptable for applications in SMG environments. As expressed in Figure 1, this paper systematically identifies limitations in fluid properties, swelling behavior, stability, and response to biochemical stimuli in hydrogels cultured under SMG and investigates how thermodynamic influences in temperature, pressure, osmotic balance, and entropy can be implemented in solutions. In this review paper, by integrating experimental findings and theoretical models, we aim to provide a framework for engineering next-generation hydrogels, optimizing their use for tissue engineering, controlled drug delivery, and space-based biomedical research.

## 2. Hydrogel in Normal Gravity and SMG

### 2.1. Normal Gravity

Hydrogels are three-dimensional, crosslinked polymer networks capable of absorbing and retaining large volumes of water, making them essential in a wide range of biomedical and engineering applications [6,20]. Under standard Earth gravity (1 g), their behavior is significantly influenced by gravity-driven fluid dynamics such as convection, sedimentation, and mass transport [8]. These forces impact critical processes during hydrogel formation and function. Convection enhances uniform solvent and nutrient distribution throughout the polymer matrix, while sedimentation affects the positioning of embedded cells or nanoparticles. Such directional forces help ensure consistent swelling behavior, controlled porosity, and homogeneous crosslinking across the hydrogel volume [6].

Gravitational forces also contribute to the development of structural anisotropy in certain hydrogel systems, which is particularly advantageous in applications like tissue scaffolding, where direction-dependent mechanical properties can support cell alignment and tissue organization [20]. Furthermore, stable gelation kinetics under gravity enable reproducible fabrication, an essential requirement for scaling up hydrogel-based medical devices or therapeutic platforms. Ultimately, gravity supports the formation of hydrogels with predictable mechanical and diffusion properties, making it a foundational consideration in the design and testing of materials for use in terrestrial biomedical systems.

#### 2.1.1. Structural and Crosslinking Mechanisms

The structural integrity and functional performance of hydrogels are largely dictated by their crosslinking mechanisms, which directly influence their mechanical strength, swelling behavior, and long-term stability [21,22]. These crosslinking strategies can be broadly classified into physical, chemical, and hybrid mechanisms. Physical crosslinking relies on non-covalent interactions—such as ionic bonds, hydrogen bonding, and hydrophobic interactions—which typically lead to reversible gelation. These gels are often stimuli-responsive but may exhibit limited mechanical strength. Chemical crosslinking, in contrast, involves the formation of covalent bonds, resulting in more robust and stable hydrogel networks. Techniques such as free radical polymerization, Schiff base reactions, and enzymatic crosslinking are commonly employed to achieve these permanent structures. To further enhance mechanical performance, dual-network and interpenetrating polymer networks (IPNs) combine two or more polymer systems. This hybrid strategy reinforces the matrix by creating a secondary network that supports and complements the primary structure. Under normal gravity conditions, the gravitational force significantly influences the gelation process. It can affect precursor mixing efficiency, alter crosslinking reaction kinetics, and introduce anisotropy in the polymer network. Additionally, shear stress induced by gravitational settling may disrupt the microstructure, impacting the porosity, uniformity, and mechanical properties of the resulting hydrogel [20].

#### 2.1.2. Fluid Behavior and Its Impact on Hydrogel Properties

Fluids play a fundamental role in determining the functional performance of hydrogels, with their behavior under normal gravity influencing hydration, structural stability, and overall responsiveness [23]. In hydrogels containing nanoparticles or embedded cells, buoyancy-driven transport significantly affects solute distribution and the formation of diffusion gradients. This is particularly relevant in applications such as controlled drug delivery and tissue engineering, where uniform solute dispersion is essential for effective function and cell viability [24]. Gravity also impacts the crosslinking process itself. During polymerization, gravity-induced phase separation can lead to inhomogeneous crosslinking and mechanical anisotropy, ultimately compromising the uniformity and mechanical stability of the hydrogel network. Studies have demonstrated that gravitational forces influence the development of hydrogel microstructures, with potential consequences for both material strength and cellular interactions [25]. Moreover, gravitational effects contribute to phase separation and sedimentation within multi-component hydrogels. This can disrupt the spatial distribution of hydrogel components, particularly in formulations with suspended particulates or gradient structures. As a result, maintaining structural uniformity and functional integrity requires careful consideration of gravitational influences during design and processing [26].

#### 2.1.3. Hydration and Swelling Behavior

Hydration and swelling are key characteristics of hydrogels, governed by a combination of osmotic pressure, polymer network architecture, and external physical forces such as gravity [20,22]. Under normal gravity, water distribution within hydrogels is shaped by the interplay of gravitational force and capillary action, which can lead to anisotropic swelling, especially in larger or layered hydrogel systems. The swelling process itself is largely diffusion-driven, occurring through Fickian or non-Fickian mechanisms, depending on the network structure and environmental conditions. Gravity can influence this diffusion process by affecting solute movement and water infiltration, ultimately altering hydration rates and swelling equilibrium [22,27]. Additionally, osmotic pressure gradients that drive hydrogel expansion may become stratified due to gravitational settling, resulting in heterogeneous swelling and localized variations in mechanical properties. Together, these factors highlight the complex interplay between fluid dynamics and gravity in shaping the structural and functional behavior of hydrogels, particularly in biomedical applications where uniformity, responsiveness, and mechanical consistency are critical.

#### 2.1.4. Mechanical Stability and Degradation

The long-term functionality of hydrogels in biomedical and industrial settings is closely linked to their mechanical stability and degradation profiles [28]. Under normal gravity, hydrogels are subject to compressive and shear forces that influence their viscoelastic behavior, including stress relaxation, creep response, and fatigue resistance. These gravity-induced loads are especially relevant in applications involving prolonged mechanical stress or load-bearing functions. Mechanical integrity is further influenced by the mode and rate of degradation. Both enzymatic and hydrolytic degradation processes are affected by gravity, which governs the diffusion of water molecules, enzymes, and other degradative agents within the hydrogel matrix. In hydrogels that degrade in bulk, gravity-driven diffusion gradients may lead to spatially uneven breakdown, compromising uniformity and structural predictability over time [29]. Additionally, gravitational forces contribute to stress accumulation at crosslinking points and interfaces within the polymer network. This can promote crack formation and eventual mechanical failure, particularly in high-stress environments such as artificial cartilage or orthopedic scaffolds. Over time, fatigue-induced damage and localized fractures can emerge under repeated loading, underscoring the importance of mechanical design considerations for durable hydrogel performance [30].

#### 2.1.5. Mathematical Modeling of Hydrogel Behavior in Normal Gravity

##### Swelling Kinetics

Swelling refers to the process in which the hydrogel absorbs a solvent and experiences an increase in volume. Osmotic force is the driving mechanism behind the swelling action, and the limiter is the elasticity force originating from the polymer’s properties. This process can be expressed as

(1) $$\frac{dV}{dt}=k(\Pi -\frac{\partial F}{\partial V})$$

Here, the swelling rate (*dV*/*dt*) is a function of osmotic pressure (*Π*), the elastic force of the polymer (*∂F*/*∂V*), and a constant derived from solvent diffusion. This process ends when the system has reached equilibrium and the forces are balanced [31].

##### Mass Transport Models

Mass transport models in hydrogels describe the transfer of substances into the gel through the processes of diffusion, convection, and osmosis. Of these processes, diffusion is the most significant. Diffusion processes can be separated into Fickian and non-Fickian transports based on the rates of solvent diffusion and polymer chain relaxation. The general model for the diffusivity of a particle is

(2) $$\frac{D_{i.s}}{D_{i.l}}=f(\xi ,\phi ,r)$$

The ratio $\frac{D_{i.s}}{D_{i.l}}$ is the diffusivity of a molecule in a solvent; *ξ* is the network mesh size; *ϕ* is the polymer volume fraction, and *r* is the diffusing molecule size. Porosity is another factor involved in the diffusivity determined by the diffusivity equation. For micro-porous hydrogels, the equation is expressed as

(3) $$\frac{D_{ip}}{D_{iw}}=(1-\lambda^{2})(1-2.104\lambda +2.904\lambda^{3}-0.95\lambda^{5})$$

The ratio here is the diffusion coefficient for a hydrogel membrane to a pure solvent, and *λ* is the ratio of solute diameter to pore size [32].

### 2.2. Simulated Microgravity

In microgravity environments, hydrogels—three-dimensional polymer networks capable of absorbing substantial amounts of water—exhibit behaviors distinct from those under Earth’s gravity. Understanding these differences is crucial for applications in space-based biomedical devices, tissue engineering, and controlled drug delivery systems. The absence of gravity eliminates buoyancy-driven effects, significantly altering polymerization, swelling, degradation, and mechanical behavior.

#### 2.2.1. Structural and Crosslinking Mechanisms

The absence of gravitational forces in microgravity environments introduces distinct effects on the self-assembly and crosslinking processes of hydrogels [20,33]. Without sedimentation and convection, polymer network formation tends to occur more uniformly, often resulting in enhanced homogeneity and improved mechanical properties compared to those formed under terrestrial conditions [34]. This uniformity is particularly advantageous in tissue engineering and drug delivery applications, where structural consistency is critical for function and biocompatibility. Crosslinking efficiency, whether through physical or chemical means, is also influenced by the microgravity environment. Gelation kinetics may be altered due to the absence of gravitational gradients, potentially modifying the rate and extent of bond formation. These changes in network development can ultimately affect the structural integrity and functional behavior of the final hydrogel [33].

#### 2.2.2. Fluid Behavior and Its Impact on Hydrogel Properties

Fluid dynamics in microgravity differ markedly from those on Earth, and these differences significantly influence hydrogel formation and function [35]. In the absence of gravity, surface tension and capillary forces dominate fluid behavior. Liquids form larger and more stable structures, and their motion is slower and more predictable, creating unique opportunities for controlled processing and observation. Microgravity also alters fluid oscillation patterns, resulting in lower-frequency sloshing behaviors. These shifts can have broad implications, especially in spacecraft design, where fluid stability is essential for maintaining attitude control and minimizing vibration-induced disturbance [24,36,37,38]. Bubble behavior is another critical difference; without buoyancy, bubbles do not rise as they do under normal gravity. Instead, surface tension governs their formation and movement, which can impact thermal management systems and complicate processes such as boiling or gas exchange [24,39,40]. Moreover, the lack of buoyancy-driven segregation reduces the natural separation of fluid phases, such as liquid–gas or liquid–liquid systems. This homogeneity poses challenges for systems that rely on clear phase boundaries, including fuel management, water purification, and life support technologies. In the context of hydrogels, this reduced phase separation can also influence the crosslinking process, as the absence of gravity-driven gradients affects how polymer and solvent components organize and react during gelation [33,39].

#### 2.2.3. Hydration and Swelling Behavior

Microgravity significantly influences the hydration and swelling dynamics of hydrogels by disrupting the conventional diffusion behavior of water molecules within the polymer matrix. In the absence of gravitational forces, hydrostatic pressure gradients disappear, leading to altered solvent transport and modified swelling kinetics compared to Earth-based conditions [1,8,41]. Studies have shown that the lack of buoyancy-driven convection under microgravity results in slower yet more homogeneous water uptake across the hydrogel network [41]. This can affect not only the time to reach swelling equilibrium but also the final volume and mechanical profile of the swollen hydrogel. In particular, swelling-induced deformations become more isotropic as directional biases caused by gravity are removed.

This isotropic expansion enhances structural predictability and may benefit applications requiring symmetrical scaffold behavior, such as load-distribution platforms and organ-on-chip models [42,43]. Additionally, microgravity alters the local hydration shell dynamics and the interplay between crosslink density and solvent accessibility, both of which can affect structural uniformity and long-term material stability [44]. These insights are crucial for the design of hydrogel-based systems that require consistent swelling performance in space or other low-gravity environments.

#### 2.2.4. Mechanical Stability and Degradation

The mechanical behavior and degradation kinetics of hydrogels are also altered under microgravity conditions [1,45]. Without gravitational stress, the mechanical integrity of hydrogels may differ, especially in contexts that involve mechanical loading or structural reinforcement. While the absence of gravity might reduce some external stressors, it can also limit the hydrogel’s resistance to strain and deformation over time, potentially affecting its applicability in load-bearing environments such as bone or cartilage scaffolds [15,16]. Degradation processes, including enzymatic and hydrolytic breakdown, are influenced by changes in fluid dynamics and mass transport under microgravity. The reduced convective flow and altered diffusion can impact the spatial distribution and activity of degrading agents, ultimately affecting the longevity and functional consistency of hydrogel-based systems in space or microgravity-SMG [8,34,46].

#### 2.2.5. Hydrogel Behavior in Normal Gravity vs. Simulated Microgravity

A comparative evaluation of hydrogel behavior under normal gravity and SMG reveals both fundamental similarities and significant differences across key functional domains. While both environments support physical and chemical crosslinking mechanisms, SMG often promotes more uniform polymer network formation due to the absence of sedimentation and convective disturbances [1,33]. Fluid dynamics also shift markedly in SMG, where surface tension becomes the dominant force, resulting in slower and more homogeneous solute distribution, in contrast to the buoyancy-driven stratification observed under Earth’s gravity [8,24,35]. These differences in fluid behavior directly impact swelling characteristics; microgravity conditions favor isotropic swelling with reduced directional bias, contributing to improved structural uniformity and predictability [41,42,43]. Mechanical integrity and degradation profiles are similarly influenced, as the lack of gravity-induced mechanical loading and altered diffusion gradients in SMG environments modify stress distribution and degradation kinetics [1,45,46]. Nonetheless, core hydrogel mechanisms such as polymer–solvent interactions, osmotic pressure regulation, and viscoelasticity remain essential in both gravitational contexts [6,21]. Table 1 summarizes these critical differences and commonalities across structural, fluidic, swelling, and mechanical behaviors. This comparison offers a consolidated understanding of how gravitational conditions modulate hydrogel functionality and serves as a foundation for the modeling strategies discussed in the subsequent section.

#### 2.2.6. Mathematical Modeling of Hydrogel Behavior in SMG

To better understand and optimize hydrogel performance in microgravity, mathematical modeling has become an indispensable tool. These models allow researchers to simulate complex behaviors such as swelling kinetics, mass transport, and phase transitions under reduced gravitational conditions [1,2]. By incorporating variables unique to microgravity environments—such as altered diffusion coefficients, capillary-driven flow, and changes in fluid–structure interactions—these simulations help predict hydrogel responses and guide the design of space-compatible biomedical materials. Modeling efforts also support the development of more accurate experimental setups and improve our ability to generalize findings from simulated to actual space environments.

##### Swelling Kinetics

In microgravity, the absence of significant gravitational forces leads to uniform solvent diffusion within hydrogels, resulting in isotropic swelling. Mathematical models based on Fick’s law and the advection–diffusion equation have been widely used to describe this swelling process, helping to predict changes in hydrogel volume and mechanical response over time [47]. These models account for the dynamic evolution of solvent uptake within the hydrogel structure, often characterized by the swelling ratio, defined as the weight of the swollen hydrogel divided by the weight of the dry hydrogel. Finite element methods (FEM) have been applied to model the swelling process in hydrogels, allowing for consideration of complex sample shapes and time-dependent boundary conditions. By integrating FEM with experimental swelling data, researchers can optimize hydrogel formulations for space applications, where uniform swelling is necessary to maintain functionality in tissue engineering scaffolds and drug delivery systems [48,49,50].

##### Mass Transport Models

Mass transport within hydrogels, including the diffusion of solvents and solutes, is significantly influenced by microgravity conditions. Models coupling mass transport with solid mechanics have been developed to characterize the behavior of hydrogels under varying environmental stimuli, such as pH and ionic strength [51]. These models integrate solvent diffusivity as an adjustable parameter to simulate transient swelling and deswelling behaviors, providing insights essential for designing advanced hydrogels for space applications. Additionally, transport models in microgravity consider the impact of reduced convection-driven mixing, which alters solute diffusion and distribution. Studies indicate that in microgravity, diffusive transport dominates over convective effects, leading to more homogeneous swelling profiles in hydrogel matrices [51]. This behavior is crucial for developing hydrogels that function reliably in space environments, such as in regenerative medicine and controlled drug delivery applications.

##### Phase Transition Predictions

Hydrogels are capable of undergoing volume phase transitions in response to external stimuli such as temperature, pH, ionic concentration, and mechanical loading. These phase changes are particularly sensitive to environmental conditions, and microgravity introduces significant alterations by modifying both thermal gradients and mechanical equilibrium [4,52]. In microgravity, the suppression of convective heat and mass transfer alters the kinetics of phase transitions, potentially slowing response times or shifting the threshold conditions for phase change. This can lead to variations in swelling–deswelling behavior and gelation characteristics, particularly in thermoresponsive hydrogels such as poly(N-isopropylacrylamide)-based systems [53]. Mechanical constraints—whether induced through confinement, loading, or dynamic actuation—can cause localized phase coexistence, resulting in heterogeneous microstructures within the hydrogel matrix. This behavior has been observed in both Earth-based and SMG conditions and is known to significantly impact mechanical performance, diffusion properties, and biological compatibility [43,54]. Phase-field models have been widely used to simulate and predict phase evolution within hydrogels.

These models consider variables such as quenching rate, system size, and mechanical loading to anticipate the formation of phase-separated regions or transient structures. For instance, Zhao and colleagues demonstrated that constrained swelling and mechanical deformation can produce complex spatial patterns of gel-rich and solvent-rich domains, influencing long-term stability and functionality [54]. Furthermore, understanding phase transitions is crucial for the design of smart hydrogels intended for space applications, where mechanical loading is minimal, but thermal and chemical gradients may still be present. Incorporating predictive modeling into hydrogel development allows for optimization of phase responsiveness, mechanical integrity, and uniformity across various gravity conditions [52,53].

## 3. Thermodynamic Principles in Hydrogel Behavior in Normal Gravity

Hydrogels, as highly hydrated polymeric networks, exhibit complex thermodynamic behaviors that govern their swelling, mechanical properties, and stability. These properties make them invaluable in tissue engineering, drug delivery, energy generation, and wound healing applications. The behavior of hydrogels under normal gravity is dictated by fundamental thermodynamic principles, including temperature gradients, pressure variations, osmotic balance, and entropy-driven interactions, which regulate crosslinking, phase behavior, and molecular diffusion [19]. Controlling these thermodynamic parameters allows for the precise engineering of hydrogel structures, optimizing their mechanical and biological functionality for biomedical applications.

### 3.1. Swelling Behavior and Thermodynamic Potentials

The swelling behavior of hydrogels is influenced by several thermodynamic parameters that dictate polymer–solvent interactions and mechanical stability. Flory’s theory describes the balance between mixing entropy and elastic retractive forces within the hydrogel network. The Flory–Huggins solution theory extends this balance by introducing an interaction parameter (*χ*) to predict hydrogel expansion or contraction. *χ* is influenced by temperature and solvent properties [31]. The swelling ratio (Volume of Swollen divided by Volume of Original) is inversely correlated to crosslink density. The quantities *φ*_2_ (the volume fraction of polymer in the swollen state), *V*_1_ (the molar volume of solvent), *ρ*_2_ (the density of the polymer), *M*_2_ (the polymer’s molar weight), and *M_c_* (the molar weight t of chains between crosslinks) are all related in the below Formula (4):

(4) $$-[ln\;ln\;\left(1-\varphi_{2}\right)+\varphi_{2}+{{\chi \varphi }_{2}}^{2}]=\rho_{2}\frac{V_{1}}{M_{c}}(1-2\frac{M_{c}}{M_{2}})({\varphi_{2}}^{0.333}-\frac{\varphi_{2}}{2})$$

An interactive graph with movable variables relating Swelling Ratio to Crosslink density can be found at this link: stevenabbott.com [55]. Under normal gravity, buoyancy-driven convection facilitates homogeneous solvent diffusion, leading to controlled swelling kinetics. Donnan’s theory governs ionizable hydrogels, regulating ionic exchange with the environment and creating osmotic pressure gradients that drive swelling behavior. In normal gravity, fluid motion enhances solute distribution, maintaining ionic equilibrium and hydrogel stability. Changes in pH and ionic strength alter the Donnan potential, affecting swelling kinetics, mechanical stability, and applications in drug delivery and biosensing. Hydrogel stability is further explained through Helmholtz and Gibbs free energy considerations. The Helmholtz free energy is applicable to constant volume systems, while the Gibbs free energy governs swelling equilibrium at constant pressure. The Gibbs free energy change during swelling can be expressed as Formula (5):

ΔG = ΔG mix + ΔG elastic(5)

where ΔG_mix represents the mixing energy of polymer and solvent, and ΔG_elastic accounts for the elastic deformation of the network. These principles guide the design of hydrogels with controlled swelling, mechanical properties, and responsiveness to external stimuli.

Recent studies have expanded upon these classical theories to include additional factors affecting hydrogel swelling. For instance, specific ion effects and the presence of multivalent ions can significantly alter swelling behavior beyond what is predicted by the Donnan equilibrium alone. Certain ions interact more strongly with polymer networks, modifying hydrogel swelling behavior. For example, kosmotropic (water-structuring) ions, such as SO_4_^(2−)^, enhance hydrogen bonding and reduce swelling, while chaotropic (water-disrupting) ions like ClO^4−^ weaken hydrogen bonds and increase swelling. These effects are not accounted for in the classical Donnan model, which primarily considers charge balance and osmotic pressure. Advanced models now incorporate these specific ion effects to more accurately predict swelling in various ionic environments [44]. Furthermore, computational simulations have been employed to study the equilibrium swelling of pH-responsive polyelectrolyte hydrogels as functions of pH and salt concentration. These simulations provide insights into the behavior of hydrogels with alternating neutral and weakly acidic blocks, enhancing our understanding of their swelling dynamics under different environmental conditions. Acidic hydrogels shrink at low pH and swell at high pH. Basic hydrogels swell at a low pH and shrink at a high pH [56].

### 3.2. Pressure Variations, Mechanical Stability, and Entropy-Driven Interactions

In normal gravity, hydrogels experience hydrostatic pressure, which affects their swelling behavior and mechanical properties. Increased external pressure compresses the polymer network, reducing solvent absorption and altering mechanical stiffness. Confinement within granular media is an example of external mechanical pressure. It can hinder hydrogel swelling due to spatially non-uniform stresses at contact points, impacting their swelling capacity and mechanical performance. Conversely, lower pressures lead to greater expansion, potentially weakening structural integrity. Microgravity environments further exacerbate these effects, as the absence of gravitational compression leads to uncontrolled swelling, which can alter hydrogel functionality in space-based applications. Load-bearing applications rely on controlled crosslink densities to maintain resilience and degradation kinetics under varying pressure conditions. Entropy-driven interactions further impact hydrogel mechanics by influencing polymer chain flexibility and relaxation dynamics. As hydrogels absorb solvent, polymer chains rearrange, leading to network expansion. Crosslink density plays a critical role, as higher crosslinking restricts swelling, enhancing mechanical strength while reducing flexibility. Entropy-modulated polymer relaxation allows for tunable elasticity, making hydrogels adaptable for various biomedical applications [19,43,44].

### 3.3. Osmotic Pressure, Ionic Transport, and Crosslinking Efficiency

The osmotic balance within hydrogels is essential for maintaining hydration and ion transport under normal gravity conditions. Osmotic gradients regulate fluid movement, affecting swelling ratios and mechanical performance. In pH-sensitive hydrogels, changes in Donnan potential influence water uptake, altering expansion behavior and stiffness. Controlled osmotic pressure is vital for hydrogel applications in tissue engineering and drug delivery, where stability and precise ion exchange are required. Crosslinking efficiency is another key factor that determines hydrogel performance. Temperature fluctuations influence gelation time, affecting mechanical integrity. Studies show that lower temperatures prolong gelation time, forming rigid polymer networks, while higher temperatures accelerate gelation, producing more flexible structures. By tailoring crosslinking density and thermal conditions, hydrogels can be optimized for specific applications requiring defined mechanical and swelling properties [6,7,44].

### 3.4. Generating Energy Through Nanofluidic Systems

Nanofluidic systems, composed of nanoscale channels embedded within hydrogels, enable selective ion transport. These systems can harness osmotic energy—electricity generated from salt concentration gradients, such as seawater and freshwater. When water flows through hydrogels with nanofluidic channels, it drives ion migration, generating an electric current through electrochemical potential differences. Recent advances in nanofluidic hydrogel membranes have significantly improved their ion selectivity and energy conversion efficiency. These membranes exploit charged polymer networks and channel architectures to enhance ion transport, achieving power densities up to 6.75 W/m^2^ under synthetic seawater and river water gradients. Moreover, the structural design of nanoconfined hydrogels has enabled cation-selective energy harvesting with power densities reaching 52.1 W/m^2^ and conversion efficiencies of up to 37.5%. These enhancements are attributed to the alignment of nanochannels and the mechanical integrity provided by crosslinked hydrogel matrices. Natural material-based nanofluidic membranes, such as those constructed from layered montmorillonite and cellulose nanofibers, have also demonstrated practical use [21,57].

### 3.5. Temperature Gradients, Phase Behavior, and Biomedical Applications

Temperature gradients significantly impact hydrogel phase transitions, where thermoresponsive hydrogels exhibit either lower critical solution temperature (LCST) or upper critical solution temperature (UCST) behaviors:

LCST hydrogels shrink at higher temperatures and swell at lower temperatures, making them suitable for thermally controlled drug release systems.

UCST hydrogels swell at higher temperatures and shrink at lower temperatures, offering applications in temperature-responsive biomaterials.

Higher temperatures increase molecular motion, accelerating solvent penetration and swelling kinetics. However, excessive heat may cause polymer degradation, reducing structural integrity and water retention. Fine-tuning temperature gradients allows for precise control over hydrogel behavior, enhancing their application in biomedical and tissue engineering platforms. For example, thermoresponsive hydrogels have been developed for minimally invasive delivery, forming gels in situ at body temperature to conform to irregularly shaped cavities, such as those resulting from traumatic brain injury. By understanding and manipulating the heat-activated phase behavior of thermoresponsive hydrogels, researchers can design materials with tailored properties for specific biomedical applications, including drug delivery systems, tissue engineering scaffolds, and responsive biomaterials [28,52,53].

## 4. Thermodynamic Principles in Hydrogel Structure Under SMG

### 4.1. Temperature Gradients and Hydrogel Behavior

Temperature critically affects hydrogel performance, influencing sol–gel transitions, crosslinking efficiency, and mechanical properties [1]. In microgravity, the absence of natural convection alters heat distribution, potentially leading to non-uniform temperature fields within the hydrogel matrix. These temperature fields can result in uneven crosslinking densities, affecting the scaffold’s structural integrity and, consequently, cell proliferation and differentiation. Maintaining controlled temperature gradients is, thus, essential for ensuring consistent hydrogel behavior in microgravity environments. National Library of Medicine’s research shows that thermoresponsive hydrogels, such as poly(N-isopropylacrylamide) (PNIPAM)-based systems, experience shifts in their lower critical solution temperature (LCST) when exposed to microgravity conditions, affecting their swelling–deswelling dynamics [53].

### 4.2. Pressure Variations and Mechanical Stability

In microgravity environments, the absence of hydrostatic pressure alters the physical dynamics within hydrogel systems, significantly impacting their swelling behavior and mechanical stability. Under reduced gravitational forces, hydrogels tend to swell more freely, leading to increased pore size and reduced mechanical stiffness [58]. This over-swelling can compromise scaffold integrity, disrupting the microarchitecture needed for proper cell attachment, proliferation, and guided migration—critical components in tissue regeneration [26]. Moreover, spatial confinement under variable mechanical stresses—such as those encountered in microgravity bioreactors or deployable tissue constructs—can further exaggerate stress inhomogeneities across the hydrogel matrix. These non-uniform stresses may result in irregular crosslinking densities and anisotropic mechanical properties. Such heterogeneity can interfere with nutrient diffusion and waste removal, negatively influencing cell viability and tissue formation. Recent studies suggest that integrating adaptive or smart hydrogel components—capable of modulating stiffness or swelling in response to local mechanical cues—can mitigate these effects. Incorporating reinforcing nanomaterials or applying secondary crosslinking strategies post-gelation can enhance mechanical resilience, providing additional control over scaffold behavior in low-pressure environments [28,43]. Understanding and compensating for these pressure-induced variations is essential for designing hydrogels suitable for long-term biomedical applications in space. This includes optimizing crosslink density, incorporating pressure-sensitive elements, and simulating microgravity conditions during scaffold fabrication and preconditioning phases [26,58].

### 4.3. Osmotic Balance and Cellular Microenvironments

Osmotic pressure plays a central role in regulating water transport within hydrogels, directly influencing their swelling behavior and the structure of the cellular microenvironment. Under terrestrial conditions, osmotic gradients help maintain hydration and ion exchange, which are vital for nutrient delivery and waste removal in tissue-engineered constructs. However, in microgravity, the absence of buoyancy-driven convection and gravitational pressure alters fluid dynamics, potentially disrupting osmotic balance [1,58]. Such disturbances can lead to excessive swelling or localized dehydration within the hydrogel matrix, resulting in heterogeneous environments that compromise scaffold uniformity and reduce cellular viability. These effects may hinder effective mass transport, impeding oxygen and nutrient diffusion while accumulating metabolic waste—conditions that are detrimental to cell growth and tissue maturation [26,44]. Furthermore, in pH-sensitive hydrogels, microgravity-induced changes in the Donnan potential may amplify osmotic imbalances, altering ion distribution and water uptake capacity [44]. This sensitivity underscores the need for precise control of osmotic gradients when designing hydrogels for long-duration space missions or orbital bioreactors. Emerging strategies to stabilize osmotic behavior include engineering hydrogels with enhanced crosslinking density, incorporating ionic buffering agents, or designing compartmentalized structures that preserve local microenvironments even under fluctuating conditions [26,28].

### 4.4. Entropy-Driven Interactions and Polymer Network Dynamics

Entropy-driven interactions—such as polymer chain flexibility, thermal fluctuations, and spontaneous network rearrangements—play a fundamental role in determining hydrogel structure, mechanics, and responsiveness. These interactions are central to the hydrogel’s ability to deform, self-organize, and mimic the dynamic nature of the extracellular matrix (ECM) [28,30]. In microgravity, the reduction in mechanical loading and absence of convection modify molecular diffusion and polymer relaxation dynamics. This shift in entropy-dominated behavior can alter the viscoelastic properties of hydrogels, influencing their ability to adapt to external forces and support cell mechanotransduction [59,60]. The decreased entropy production in such environments can result in slower relaxation rates, affecting hydrogel remodeling and responsiveness over time. These changes may directly impact critical biological functions—such as stem cell fate, intercellular signaling, and mechanosensitive gene expression—especially in hydrogel systems intended to replicate ECM environments for space-based regenerative medicine [18,59]. To address these challenges, researchers are developing hydrogels with tunable viscoelasticity and dynamic crosslinking mechanisms that respond predictably in low-gravity settings. For instance, hydrogels that exploit reversible supramolecular bonding or temperature-sensitive relaxation pathways offer avenues to restore entropy-driven adaptability under microgravity conditions [28,30]. In the Table 2 we demonstrated a Comparison of thermodynamic parameters in hydrogel behavior under normal gravity and microgravity. Understanding and engineering these entropy-governed behaviors are essential for designing next-generation hydrogel scaffolds that maintain their biological functionality across different gravitational environments.

### 4.5. Thermodynamics as a Framework for Standardizing Hydrogel Behavior in Microgravity

The absence of standardized methodologies in hydrogel research under microgravity remains a critical barrier to progress. Variability in simulation techniques, hydrogel formulations, and biological assessment criteria leads to inconsistent outcomes that hinder reproducibility and cross-study comparisons. This challenge is exemplified by the contrasting findings of Buravkova and Jeyaraman. Buravkova et al. observed that in SMG, osteoblasts exhibited decreased osteocalcin and collagen gene expression, indicating impaired extracellular matrix (ECM) production and bone differentiation potential [59]. In contrast, Jeyaraman et al., using chondrocyte cultures aboard the International Space Station, reported enhanced ECM synthesis, increased expression of SOX9 and collagen type II, and improved tissue architecture under true microgravity conditions. Interestingly, their simulations using rotating wall vessels and random positioning machines reproduced some effects, such as spherical cell morphology and SOX9 upregulation, but failed to replicate the full profile of differentiation and cytoskeletal remodeling observed in space [60]. These discrepancies underline the significant impact that differences in both gravity simulation methods and hydrogel systems can have on cellular responses. For example, material properties such as crosslinking density, stiffness, and osmotic responsiveness are often inadequately reported or standardized, yet they are known to strongly influence cell behavior in 3D cultures [28,30]. To bridge this gap, thermodynamic parameters—such as swelling pressure, Donnan potential, and sol–gel phase transitions—can provide a unifying framework for hydrogel characterization. Quantitative modeling of hydrogel behavior based on these parameters allows for consistent measurement and prediction of physical and biological responses across different experimental setups, including those in altered gravity [44,52]. Incorporating thermodynamic analysis into experimental design will not only enhance reproducibility but also accelerate the translation of hydrogel systems into real-world space medicine applications.

## 5. Conclusions

**Figure 2:**
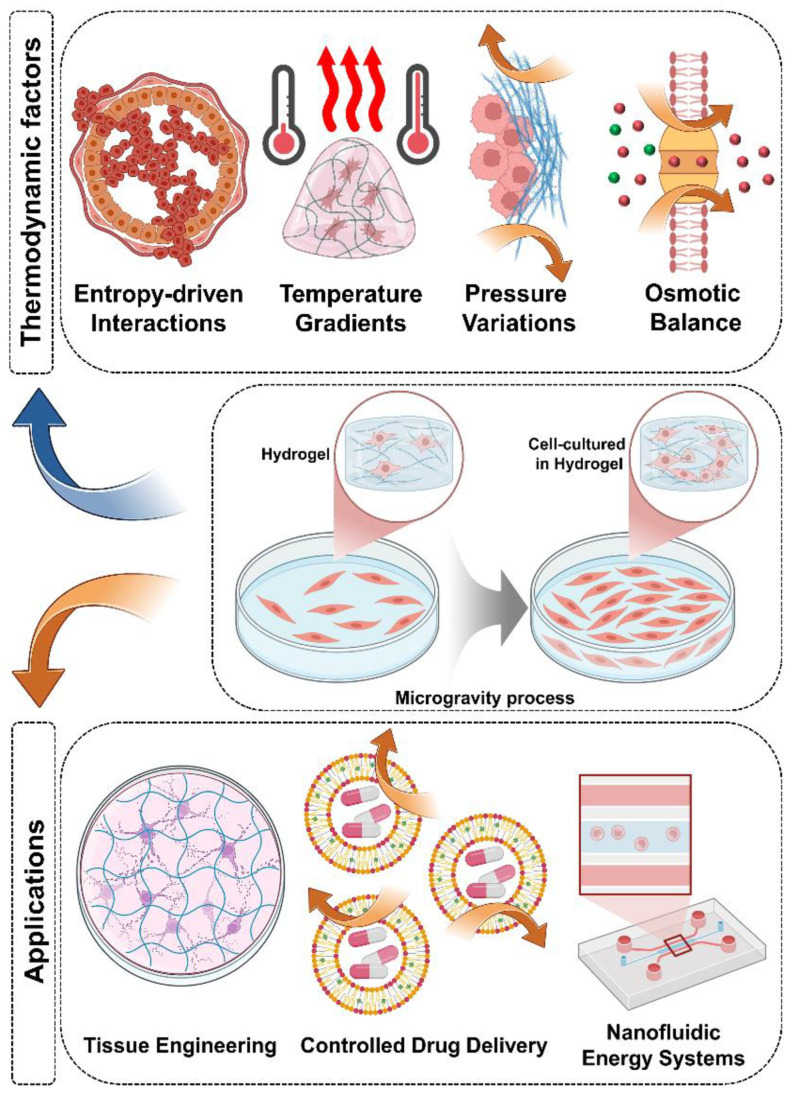
Thermodynamic strategies to counteract microgravity-induced alterations in hydrogel behavior, highlighting key challenges and corresponding stabilization mechanisms.

Hydrogels are foundational materials for advancing tissue engineering and biomedical systems in space due to their tunable mechanics, responsive swelling behaviors, and inherent biocompatibility. However, their performance is profoundly influenced by environmental conditions—especially under SMG—which can disrupt structural integrity, transport dynamics, and network stability. This paper provides a comprehensive comparative analysis of hydrogel behavior under normal gravity versus SMG, offering both physical interpretations and thermodynamic insights into how factors such as polymer crosslinking, solute diffusion, and matrix evolution are affected. A central contribution of this work is the establishment of a thermodynamic framework that defines and contextualizes hydrogel behavior in varying gravity conditions. Key parameters—such as temperature gradients, osmotic pressure, pressure differentials, and entropy-driven swelling—were systematically analyzed. These variables were shown to be critical in mitigating issues commonly encountered in low-gravity environments, such as non-uniform crosslinking, anisotropic deformation, and instability during degradation. Our findings indicate that by tuning these parameters, hydrogel systems can be optimized for functional reliability in both microgravity and terrestrial biomedical platforms. Importantly, this framework is strengthened through the integration of mathematical modeling strategies. Finite element simulations, phase-field modeling, and kinetic swelling equations were incorporated to predict phase transitions, deformation patterns, and fluid transport under dynamic conditions. These models provide a powerful predictive toolset, allowing researchers to simulate the effects of thermodynamic adjustments before physical implementation. By doing so, the modeling not only supports theoretical exploration but also informs experimental design—bridging the gap between concept and application. Beyond its scientific insights, this paper addresses the urgent need for methodological standardization in hydrogel research under microgravity. Discrepancies observed in studies by Buravkova and Jeyaraman underscore how variability in SMG simulation and hydrogel characterization techniques can lead to conflicting results. The section on standardization outlines the need for unified protocols in materials preparation, exposure conditions, and outcome assessment. Without this, direct cross-study comparisons will continue to be compromised, hindering progress in the field. Figure 2 of this work serves as a visual synthesis, mapping the relationship between thermodynamic parameters and hydrogel performance under SMG. It acts as both a conceptual framework and a practical guide for engineers and scientists designing next-generation hydrogels for use in altered gravity environments.

While the framework presented offers a solid foundation, experimental validation in true microgravity settings (e.g., aboard the ISS) remains a key future goal. Developing real-time, in situ monitoring systems to assess hydrogel behavior dynamically, along with integrating adaptive bioactivity and mechanical resilience, will be critical to the success of hydrogel technologies in space. In conclusion, this study lays the thermodynamic, comparative, and modeling groundwork for understanding and engineering hydrogel systems optimized for microgravity.

By combining predictive thermodynamic modeling with experimental insight and addressing the need for standardized methodology, this work contributes a cohesive platform for the precise design and deployment of hydrogels—on Earth and beyond.

## Figures and Tables

**Table 1:** Comparative Overview of Hydrogel Behavior in Normal Gravity and SMG.

Aspect	Normal Gravity (1 g)	Simulated Microgravity (μg)
Crosslinking Mechanisms	Crosslinking polymers of hydrogels exist in many forms, such as chemical covalent bonds, and physical bonds, such as ionic and hydrogen bonds. These mechanisms are affected by sedimentation due to gravitational forces and natural convection, which may lead to anisotropy [22].	The microgravity environment results in a lack of natural convection and removes the driving force for sedimentation, resulting in more uniform crosslinking of polymers. This is especially so in the physically bonded polymer crosslinks. The result is improved strength and rigidity, but at the cost of flexibility [13].
Fluid Behavior	Under normal gravity, buoyancy drives the transport process within the hydrogel. Phase separation also occurs during swelling in the polymer network. This can result in uneven solute distribution [22].	In the absence of gravity and capillary forces, surface tension effects will be dominant. This results in larger, more stable bubbles and homogeneous mixtures, leading to increased fatigue resistance and reduced swelling behavior [23,24].
Swelling Behavior	The swelling behavior of a hydrogel will be anisotropic as it attempts to reach equilibrium due to the forces of gravity and capillary action [24].	Without the forces of gravity and capillary action acting on the hydrogel, the material will exhibit much more uniform swelling behavior. The advantages of uniform swelling are a more stable structure and minimized stress points [24].
Mechanical Stability	Due to the composition of hydrogels, they exhibit mechanical properties of both viscous and elastic materials. Under a stressor such as the force of gravity, the material undergoes fatigue and creep [22].	With minimal external stresses, the hydrogel can experience increased amounts of swelling while potentially experiencing higher stability through less fatigue/creep.
Degradation	Degradation of a hydrogel involves change in properties due to breakdown of the polymer structure. This is often achieved through the diffusion of an enzyme into the material. In a normal gravity environment, this diffusion can be uneven, resulting in a comparably uneven breakdown [22].	In SMG, a more uniform degradation can be expected, potentially slowing the overall process. This is beneficial because it leads to a predictable loss of mechanical properties and a controlled release rate.

**Table 2:** Comparison of Thermodynamic Parameters in Hydrogel Behavior Under Normal Gravity and Microgravity.

Thermodynamic Parameter	Normal Gravity (1 g)	Microgravity (µg)
Temperature Gradients	Heat Transfer: Natural convection facilitates uniform heat distribution within hydrogels, promoting consistent crosslinking and mechanical properties. Thermal Conductivity: Standard temperature regulation ensures well-defined sol–gel transitions and polymer relaxation [52].	Heat Transfer: Absence of convection leads to reliance on conduction and radiation for heat transfer, potentially causing non-uniform temperature distribution. Thermal Conductivity: Altered thermal properties may affect hydrogel stability and performance. Phase Transition: It can be concluded that reduced gravity conditions may shift sol–gel transition thresholds and hydrogel response times.
Pressure Variations	Hydrostatic Pressure: Standard atmospheric pressure maintains typical swelling ratios and mechanical stability. Mechanical Properties: Hydrogels exhibit predictable compressive strengths suitable for load-bearing applications [61]. Network Stability: Consistent pressure conditions support uniform polymer crosslinking and mechanical resilience [62].	Hydrostatic Pressure: Reduced pressure can lead to increased swelling, altering pore size and mechanical strength. Mechanical Properties: Changes in pressure may affect the structural integrity of hydrogels in microgravity environments. Network Stability: Variations in mechanical load distribution can cause unpredictable deformations in hydrogels [63].
Osmotic Balance	Solvent Movement: Gravity-driven convection aids in maintaining osmotic equilibrium. Swelling Behavior: Controlled osmotic pressure supports stable swelling and deswelling cycles. Ion Transport prevents pH fluctuations [58]. Water-structuring ions enhance hydrogen bonding and reduce swelling, while water-disrupting ions weaken hydrogen bonds and increase swelling [44]. Osmotic energy harvesting creates an electric current through concentration differences [57].	Solvent Movement: Lack of convection may disrupt osmotic balance, uneven swelling, and dehydration. Swelling Behavior: Altered osmotic conditions can affect hydrogel performance in microgravity. Ion Transport: Reduced buoyancy-driven mixing may lead to heterogeneous ion distributions [58]. Microgravity can encourage water to move to a higher concentration, yielding higher energy from osmotic energy harvesting [57].
Entropy-Driven Interactions	Polymer Chain Dynamics: Entropy influences polymer flexibility and network rearrangements, affecting hydrogel elasticity and resilience. Molecular Diffusion: Standard diffusion rates facilitate predictable drug release profiles. Phase Separation: Thermodynamic stability ensures proper distribution of polymer chains, avoiding microstructural defects [44,55]. In situ delivery, an entropy-driven reaction, uses the heat-activated phase behavior of thermoresponsive hydrogels to tailor materials for specific biomedical applications, including drug delivery [5].	Polymer Chain Dynamics: Microgravity may prevent polymer relaxation and hydrogel mechanical properties. Molecular Diffusion: Changes in diffusion rates could affect nutrient transport within hydrogels. Phase Separation: Lack of gravity-driven segregation results in more homogeneous hydrogel structures [44,55].
pH	Acidic hydrogels shrink at low pH and swell at high pH. Basic hydrogels swell at low pH and shrink at a high pH [56].	The effect of pH on swelling is unchanged by microgravity [56].

## Data Availability

No new data were created or analyzed in this study. Data sharing is not applicable to this article.
